# Carbohydrate Reward and Psychosis: An Explanation For Neuroleptic Induced Weight Gain and Path to Improved Mental Health?

**DOI:** 10.2174/157015911795596513

**Published:** 2011-06

**Authors:** Simon Thornley, Bruce Russell, Rob Kydd

**Affiliations:** 1 Section of Epidemiology and Biostatistics, The University of Auckland, Private Bag 92019, Auckland, New Zealand; 2 School of Pharmacy, Faculty of Medical & Health Sciences The University of Auckland, Private Bag 92019, Auckland, New Zealand; 3 Clinical Psychological Medicine Faculty of Medical & Health Sciences The University of Auckland, Private Bag 92019, Auckland, New Zealand

**Keywords:** Antipsychotic agents, addictive behaviours, glycemic index, carbohydrates.

## Abstract

Evidence links dopamine release in the mid-brain to the pathophysiology of psychosis, addiction and reward. Repeated ingestion of refined carbohydrate may stimulate the same mesolimbic dopaminergic pathway, rewarding such eating behaviour and resulting in excessive food intake along with obesity. In this paper, we explore the role of dopamine in reward and psychosis, and discuss how reward pathways may contribute to the weight gain that commonly follows antipsychotic drug use, in people with psychotic illness. Our theory also explains the frequent co-occurrence of substance abuse and psychosis. From our hypothesis, we discuss the use of carbohydrate modified diets as an adjunctive treatment for people with psychosis.

## BACKGROUND

The mesolimbic dopaminergic system of the mid brain is intimately linked to two major categories of disease (1) addiction, and (2) psychotic disorders. Although, the link between mental illness and addiction has been commonly reported [1], with increased prevalence of substance abuse in people with psychosis, the theoretical links between these disorders has not been explored in detail. In this article, we argue that psychosis and addiction are closely linked, sharing similar anatomical regions of the brain which demostrate dysfunction in both syndromes [1]. Because of such common features, we speculate that reducing the use of addictive substances in people with mental illness is likely to improve their underlying mental disorder. By achieving similar neurophysiological effects (less dopaminergic stimulation), reducing addiction is likely to complement the effect of antipsychotic drugs. Along with substance abuse, we argue that food intake shares clinical features of addiction; especially the consumption of sugars and refined carbohydrates. We first consider theoretical and observed links between psychosis and addiction, then postulate a new hypothesis, which may explain the weight gain that commonly occurs after antipsychotic drug use [2-5]. An important suggestion arising from this theory - that carbohydrate modified diets may improve the mental health of people who suffer from psychotic symptoms – is then outlined.

To explore the intersection of psychotic and addiction phenomena, first, we briefly define addiction, along with its clinical features. Next, we review the neuroanatomy and physiology of the dopaminergic pathways in the midbrain, and how this region behaves in both disorders. We then discuss the mechanism of action of antipsychotic drugs and new evidence linking food intake and obesity with addiction. Our hypothesis then links psychosis, food and addiction and we explain how this may offer new dietary therapy for people with schizophrenia.

## ADDICTION

When does a person’s substance use cross the boundary to become an addiction? Controversy surrounds the definition, with different conceptions offered. Heather describes addiction as “*repeated failures to refrain from drug use despite prior resolutions to do so*” [6]. The DSM –IV [7] criteria expand on this definition, requiring three or more of the following criteria occurring within a twelve month period: (1) taking larger amounts of the substance than intended; (2) unsuccessful efforts to cut down; (3) a great deal of time devoted to acquiring the substance; (4) giving up important activities because of substance use; (5) investing a great deal of time in acquiring or using the substance; (6) continued use despite negative consequences; (7) increased need for the substance indicating tolerance; and (8) withdrawal manifest by a characteristic syndrome if the substance is withdrawn or the substance is taken to avoid such discomfort.

The last feature of the DSM-IV criteria, the withdrawal syndrome, may be the most important, given the subjective nature of the other features. Withdrawal symptoms include depressed mood, disturbed sleep and a wide variety of somatic symptoms [8] and may often be subtle, described by recovering users as “just not feeling right”. Substance use relieves this discomfort, resulting in negative re-inforcement of the action with repeated drug use to avoid the unpleasant symptoms [9]. Opiate withdrawal, for example is characterised by severe somatic symptoms including stomach cramps, intense craving, aches and pains, sweating and palpitations [10]. A contrasting cluster of symptoms is seen in tobacco withdrawal: notably craving and urges to smoke, irritability, reduced concentration, increased appetite and constipation [11]. Although the nature of these withdrawal states may be quite different, the time course over which the symptoms are experienced is roughly consistent, with the intensity peaking after three to four days, then gradually declining after one to three months of abstinence.

## PHYSIOLOGY OF ADDICTION

In the last thirty years the origin of clinical symptoms of addiction has been linked to a part of the brain responsible for subconscious behaviour and motivation, and a specific neurotransmitter: dopamine. The dopaminergic system is composed of two major pathways: (1) the nigrostriatal system that projects from the substantia nigra in the basal ganglia to the corpus striatum and (2) the mesocorticolimbic dopamine system, which emanates from the ventral tegmental area of the mid brain to the nucleus accumbens and olfactory tubercle (in the ventral striatum), medial pre-frontal cortex and amygdale (found in the temporal lobe) [12]. These neural pathways each have their own functions, which have been understood by their association with the symptom clusters that accompany disease states in their respective anatomical regions. The mesocorticolimbic projection is most often implicated in the pathophysiology of addiction and subconscious reward pathways. In human and animal studies, administration of substances of abuse increases dopamine concentrations in the nucleus accumbens, considered the main component of the brain reward system [12]. An intravenous bolus of cocaine, for example, causes increased extracellular concentrations of dopamine, blocking re-uptake by nerve terminals in the nucleus accumbens. In contrast, opioids, nicotine and alcohol act upstream, stimulating neurones in the ventral tegmental area, which ultimately influence the nucleus accumbens, by releasing dopamine at nerve terminals into the synaptic cleft and activating the nucleus. These three drugs either act directly on the cell bodies of these ventral tegmental neurones or block inhibition by GABA interneurones [13].

Also, extracellular dopamine release in the striatum (from psychostimulants) and subjectively feeling “high” or euphoric [14] are closely linked, in human brain imaging studies. Although dopamine is often described as the “pleasure chemical”, this role is currently debated. Some suggest that dopamine release in the nucleus accumbens has less to do with reward, but is more likely to reinforce, strengthen and initiate movement required to attain the eventual reward, such as rolling a cigarette [15].

The nigrostriatal pathway, which includes the substantia nigra, is best known for its relationship to the degenerative disorder, Parkinson’s disease, characterised by a cluster of motor and non-motor symptoms. Motor symptoms include a characteristic ‘pill-rolling’ tremor, shuffling gait, brady-kinesia (slowing of motor movements), and rigidity. Non-motor manifestations consist of depressed mood, cognitive impairment along with autonomic and sleep disturbances. Replacement of dopamine, in precursor form (levodopa), partially relieves many of these symptoms. Degeneration of the dopaminergic neurones in this part of the brain are seen in post mortem studies of people who have died with Parkinson’s disease [16].

## PATHWAYS OF ADDICTION TO FOOD

The mesolimbic dopaminergic pathway is activated by drugs and substances that people ingest, inject, snort or smoke that, ultimately, cross the blood brain barrier, after entry into the circulation. Over the last two decades, other evidence has implicated this pathway in weight control and eating behaviour. This evidence comes from a number of different studies. These include microdialysis, rodent based research of the mesolimbic pathway that have shown that exposure to food results in dopamine release. Although the quantity is less than what occurs in response to drugs of abuse, such as amphetamines [17]. The magnitude of dopamine release, in rodent models, is about 10 fold greater with a more rapid rate of initial rise, after an amphetamine bolus, compared to the release which follows a meal, mixed in macronutrients [18].

It is also recognised that the brain’s primary fuel in the non-fasting state is glucose, and changes in venous glucose concentrations trigger changes in the firing of dopaminergic neurones in the striatum [19]. Other studies point to a direct physiological effect of plasma glucose on dopamine release in neuronal tissue. For example, Koshimura incubated nerve cells in high concentrations of glucose, and found that depolarisation and dopamine release was enhanced [20]. In addition, rodent studies have demonstrated that restricted feeding coupled with intermittent sucrose availability leads to up-regulation of the dopamine transporter in the nucleus accumbens and ventral tegmental area [21]. Sucrose consumption has also reliably induced signs of addiction in rodents, with demonstrated anxiety and somatic symptoms (withdrawal) following abstinence, or after naloxone (an opiate antagonist) administration [4]. Although high fat rodent diets also stimulate dopamine release in the nucleus accumbens, similar withdrawal symptoms were not observed when these animals were forced to abstain or treated with naloxone [22]. In humans, carbohydrate craving has often been reported, although a full withdrawal syndrome has not yet been described. We portrayed one individual who demonstrated a likely food withdrawal syndrome following abstinence from sugar and carbohydrate [23]. Another example is given by Atkins, in his well known book, portraying an obese individual who suffers from craving and agitation relieved by sugar, that eventually resolves within weeks after starting his carbohydrate restricting diet [24].

Tolerance or need for an increased quantity of a substance to get the same ‘hit’ is a dominant feature of addiction and may reflect reduced sensitivity to dopamine by post-synaptic neurones in the nucleus accumbens. Anatomical changes found following Positron Emission Tomography studies of those who suffer from drug addiction show evidence of adaptation, with an increased concentration of dopamine receptors compared to controls [25]. Such changes in the accumbens also occur in obese individuals [26].

Of all the food groups, carbohydrate is commonly ascribed addictive properties [27], and within this class, table sugar or sucrose [28]. This disaccharide is composed of two chemically linked monosaccharides: glucose and fructose. Glucose is the primary energy source of the brain and other vital organs, compared to fructose which has stronger sensory qualities, percieved as about twice as sweet as similar concentrations of glucose. Fructose, unlike glucose, is taken up preferentially in the liver, and so, is almost absent in peripheral blood [29].

The mechanism by which sucrose induces dopamine release is uncertain, however, two possibilities are commonly cited [30]. First, sugar may be ingested, absorbed and the glucose transported to the brain from the circulation, with subsequent dopamine release. Second, direct sensory input from taste and other sensory input in the mouth may provoke dopamine release. When rodents are “sham fed” – allowed to feed, but not digest food by having it pass out of the body *via* a gastric fistula - concentration-dependent dopamine release follows [30]. Further release of dopamine occurs during digestion and absorption of glucose, which is potentiated by glucose-mediated insulin release [31]. Such findings reiterate why fructose may be an important contributor to food addiction, owing to its sweeter taste. Importantly, fructose (unlike glucose) does not appear to stimulate insulin release, a hormone associated with post-prandial satiety.

What are the clinical features of drug induced dopamine release in human subjects? Two principal effects are commonly described [13]. First, release is accompanied by pleasure, or the feeling of a ‘hit’ and behaviours that produce this are reinforced, forming a vicious cycle of increasing substance use. In addition, the increase in dopamine concentration focuses the individual on sensory elements (‘cues’) associated with drug taking. Such elements often initiates subconscious, Pavlovian stimulus-response drug taking when an individual is subsequently exposed to them again [13].

## NEURAL PATHS ASSOCIATED WITH PSYCHOSIS

As well as playing a role in addiction, dopamine has been referred to as the “wind of the psychotic fire”, when describing its association with the pathophysiology of schizophrenia [32]. Evidence for dopamine's importance emerges from clinical practice - for example, treatment of patients with Parkinson’s disease using therapeutic doses of levo-dopa can result in a drug-induced psychosis in a small proportion of these patients [33]. Conversely, drugs used to treat psychoses such as schizophrenia inhibit dopaminergic pathways, and may result in unwanted Parkisonism.

Antipsychotic or ‘neuroleptic’ drugs have a specific mechanism of action. After entry into the circulation they diffuse across the blood-brain barrier. There, they bind to and block neural circuits, specifically interfering with the action of dopamine on mesolimbic and mesocortical D_2_ receptors [34]. Greater specificity for the blockade of serotonin receptor subtype 5-HT_2A_ is a feature of atypical antipsychotic agents, such as clozapine and olanzapine. Although dopaminergic dysfunction is highlighted most frequently in the pathophysiology of psychosis, other neural paths are also implicated, such as the glutaminergic and serotonergic [35] ones.

Given the association between dopaminergic neural paths and addiction and psychosis, at a clinical level we may expect a close relationship between the two disorders. For example, in one summary, the prevalence of smoking was between 80 and 90% in people treated in hospital with schizophrenia [1]. Numerous epidemiological studies describe the co-occurrence of schizophrenia and other forms of addiction, such as to alcohol, metamphetamine and opiates. [1]. Further, the presence of illicit drug use in people with schizophrenia predicts relapse, treatment resistance and need for further hospital treatment [1].

Researchers have concluded that no drug with significant antipsychotic action has been identified that does not have a significant affinity for the D_2_ receptor [36]. In the spinal fluid of psychotic patients, maintained on a variety of 11 different antipsychotic drugs, seventy percent blockade of the D_2_ receptor *in vitro* correlated to therapeutic free neuroleptic levels [36]. The altered side effect profiles attributable to newer antipsychotic agents have been linked to their briefer occupancy of the D_2_ receptor site (in contrast to older agents, which bind for longer) along with increased serotonergic effects.

For a long time, activity in the dopaminergic system has been difficult to directly assess in human subjects, but recently, positron and photon emission tomographic techniques have enabled this pathway to be studied in detail. Several connections between dopamine and psychosis are described. First, amphetamine induced dopamine release is exaggerated in people with schizophrenia; second, after amphetamine administration, dopamine release is associated with psychotic symptoms; and third, these changes in dopaminergic physiology in people with schizophrenia have been detected in patients never treated with antipsychotic drugs [32]. This last finding indicates that such phenomena are unlikely to represent neuroadaptation to antipsychotic medication.

What is the consequence of excess ‘reward’ or ‘re-inforcement’ from dopamine release? Recent theories suggest that in people with schizophrenia, dopamine release leads to aberrant salience or focussing on innocuous stimuli [37]. Symptoms of hallucinations and psychosis emerge, resulting from a patient’s attempt to explain the increased attention they give to such stimuli. This mechanism may also explain the negative symptoms of schizophrenia, such as social withdrawal and lack of motivation. The increased “noise” in the reward pathway, due to aberrant dopamine release, may drown out the normal linkage and learning that accompanies behaviours that lead to reward from dopamine release [37].

## HYPOTHESIS

If dopamine is the principal neurotransmitter mediating both re-enforcing behaviour and reward from ingestion of food, what are the likely consequences of blocking this action with antipsychotic drugs? Our hypothesis links the increased prevalence of obesity found in people treated with anti-psychotic agents and the known pharmacodynamic properties of such drugs - blocking dopamine effects in the reward centre of the mid-brain. We speculate that to compensate for the reduced reward after taking neuroleptics, patients exaggerate the stimulation of this pathway by increasing their food intake, or use of other addictive substances.

## SUPPORT FOR THIS HYPOTHESIS

Rodent studies have supported our hypothesis that antipsychotic drug use enhances feeding behaviour. In general, long term exposure of rodents to antipsychotic agents is associated with weight gain [4], although some short term studies show inconsistent results. The most conclusive study showed both a dose response effect (increased weight gain with increased dose), and reversal of this effect with bromocriptine, a specific dopamine (D_2_) receptor agonist [4]. Four antipsychotic drugs consistently produced weight gain (thioridazine, trifluoperazine, haloperidol, and sulpiride), whilst chlorpromazine and fluphenazine did not. Another study documented increased food consumption in rodents treated with olanzapine, an atypical anti-psychotic medication, compared to untreated controls [38].

In human subjects, weight gain commonly occurs in association with antipsychotic use, however the mechanism underlying this effect is not well understood. Several explanations are proposed, including increased appetite, leading to excess energy intake [3]. Metabolic changes, which lead to reduced metabolic rate, such as insulin resistance, have also been described [3, 39]. For example, weight gain has been consistently reported with clozapine – a retrospective study of 82 patients, followed up to 90 months, showed a cumulative incidence of a 10% weight gain in 80% of subjects, with a 20% weight gain observed in 38% [40]. Many other studies have confirmed such findings and some authors link weight gain with clinical improvement in mental state [41, 42]. The mechanism underlying these changes in weight has remained uncertain, with a number of neurotrans-mitters and hormones implicated [2] within serotonergic, histaminergic and adrenergic pathways [3].

We speculate that if dopaminergic neural transmission is blocked by antipsychotic drugs in patients with schizophrenia, compensation may occur, with patients adapting by carrying out behaviours that stimulate dopamine release (from food or drug intake) in an effort to maintain homeostasis, achieving the same feeling of reward, satisfying hunger or drug withdrawal symptoms. Evidence for such an effect can be found in animal experiments which demonstrate dulling effects of antipsychotic drugs on chemical or electrical stimulation of mid- brain reward paths [17]. Also, animal studies of amphetamine and cocaine self administration show that short term compensatory increases occur after neuroleptic treatment [43]. An older study showed that the rewarding qualities of food were blocked by neuroleptics in hungry rats [44]. Although many recreational drugs induce dopamine release, the ubiquity of carbohydrate containing food may make this the most common means of compensating for the action of antipsychotic drugs. People with schizophrenia often, however, may use other drugs to compensate for dopamine blockade, and indeed substance (cocaine, cigarette, alcohol, methamphetamine) abuse and psychosis commonly coexist.

Our theory focuses on the centrality of the dopamine receptor blockade of antipsychotic drugs and their simultaneous effects on weight gain and psychosis. One potential flaw in this theory is the differential effects on weight gain of different drugs. For example, in studies in which patients are followed for less than one year of treatment, a clear hierarchy of weight gain liability occurs, with clozapine>olanzapine>risperidone>ziprasidone=aripiprazole [5]. Although all of these drugs block D_2_ receptors, they vary substantially in the amount of serotonergic, histaminergic and adrenergic activity they exhibit. Such a finding may indicate that other pathways or neurotransmitters are more likely to be causally implicated in weight gain side effects than the dopaminergic path. However, in studies which observe weight gain in patients treated for more than one year, such differences between drugs are less marked, and some of the between drug heterogeneity may be explained by between-study variation in dosing [45].

## IMPLICATIONS

How might our theory improve care for people with psychosis? We speculate that if addiction and psychosis share common features and a common neuroanatomical pathway, with dopamine release and disordered reward and motivation, a key treatment for reducing the severity of psychosis may involve reducing stimulation of the mid brain. If antipsychotic drugs exert their effect, principally, by blocking the effects of mesolimbic dopamine release, we expect that reduced release of dopamine in this pathway may result in similar beneficial effects on psychotic symptoms in people affected by schizophrenia. Although still controversial, we speculate that food may be a key stimulant of this disordered pathway, and altering diet may improve psychosis and reduce the need for antipsychotic treatment. If blocking the effects of free dopamine reduces psychotic symptoms, then reducing dopamine release is likely to induce a similar effect. Offering treatment for other addictions (not only food) to limit dopamine release also follows from our theory.

Surprisingly, evidence for nutrition interventions to improve psychotic symptoms has received little attention. Peet speculated that the co-occurrence of schizophrenia and diabetes, even in drug naive patients, suggests that the two disorders share a common aetiology [46]. One study reports high levels of sugar consumption in patients with schizophrenia, although the authors are uncertain whether this is a cause or consequence of treatment for the disease [47]. Dietary treatment for patients with schizophrenia usually consists of reducing long term cardiovascular and diabetes risk, rather than being viewed as an integral part of reducing psychotic symptoms. To our knowledge, the effect on psychopathology of reducing sugar or carbohydrate consumption among a cohort of people with schizophrenia has not been studied.

If a dietary approach to treating psychotic symptoms was suggested, what might this regime look like? Glycemic index (GI) describes the rate of absorption of glucose from carbohydrate. High GI carbohydrate foods, such as white bread, sugar and refined cereals, cause a ‘spike’ in insulin and blood glucose (followed by a dip). Low GI carbohydrate foods, such as wholegrain bread and most vegetables (or foods containing little carbohydrate at all, such as nuts, seeds and meat), produce a slower insulin response and thus a more steady supply of glucose to the blood. Low GI diets induce weight loss and improve blood glucose control in people with diabetes. A recent meta-analysis showed that people following a low GI diet had more weight loss than other diets [48]. Other meta-analyses indicate a lower risk of chronic diseases such as cancer and cardiovascular disease occur in people following low GI diets [49]. We speculated that one indicator of the addictive potential of food is the glycaemic index, based on the principle that ‘time-to-reward’ (or in pharmacokinetic terms, rate of rise of plasma concentration) predicts the addictive potential of a substance [50]. Although this scale discounts the importance of sugar content, due to the presence of fructose which has little effect on blood glucose. The two best known diets based on modifying carbohydrate consumption include the glycaemic index and the Atkins diet. Low GI diets emphasise the selection of foods with a low to moderate index (<55%) [51]. The Atkins diet is more strict, with very low carbohydrate consumption (<20g/day) in the initial phases, with a graduated re-introduction of this food group after initial weight loss [24]. Studies of the effects of low glycaemic index diets on dopamine release are yet to be carried out, however anecdotal reports suggest an initial discomfort and craving after starting this regime, which suggests a component of withdrawal.

Studies that measure the effect of carbohydrate modified diets on psychotic symptoms would test whether our hypothesis is tenable. Drop out and compliance with carbohydrate restricted or modified diets is often low, and engagement of people with schizophrenia in such treatment may be more complicated than those which involve obese people. Nevertheless, a non-pharmacological approach to the treatment of psychosis may be more acceptable than drug treatments that are often associated with unpleasant side effects and low levels of adherence to treatment. Carbohydrate modified diets may also provide an adjunct to antipsychotic medication, potentially limiting unintended effects such as weight gain and adverse increases of other indices of cardiovascular risk. Meta-analyses of low glycaemic index diets show that these interventions are effective for achieving sustained weight loss in obese people [31] and reducing the risk of a range of chronic diseases such as such as type 2 diabetes, coronary heart disease, gallbladder disease, breast cancer and all diseases combined (Rate Ratio 1.14, 95% CI 1.04 to 1.15) [52]. Given the increased mortality associated with schizophrenia [53-55], use of these diets among people with psychotic illness may achieve a reduction in the inequity of physical health status and longevity compared with people without such conditions.

Our hypothesis also reinforces the rationale for treatment of addictions in people with schizophrenia. The use of short-term nicotine replacement therapy and quit advice, limitation of caffeine intake, and treatment of methamphetamine, alcohol and other substance abuse in such patients are likely to not only improve the patient’s general health, but also assist their recovery from psychosis.

## CONCLUSION

Our theory provides a parsimonious and testable hypothesis, linking the action of antipsychotic agents with commonly reported side effects. It also explains the common co-occurrence of schizophrenia with addiction, obesity and diabetes. The common link drawn between eating, psychosis and mid-brain dopaminergic reward, logically, suggests that psychosis may be improved, by modifying carbohydrate consumption. We consider that such an idea should be tested in clinical trials.
